# Inpatient or Outpatient Rehabilitation after Herniated Disc Surgery? – Setting-Specific Preferences, Participation and Outcome of Rehabilitation

**DOI:** 10.1371/journal.pone.0089200

**Published:** 2014-03-05

**Authors:** Margrit Löbner, Melanie Luppa, Alexander Konnopka, Hans J. Meisel, Lutz Günther, Jürgen Meixensberger, Katarina Stengler, Matthias C. Angermeyer, Hans-Helmut König, Steffi G. Riedel-Heller

**Affiliations:** 1 Institute of Social Medicine, Occupational Health and Public Health, University of Leipzig, Leipzig, Germany; 2 Department of Health Economics and Health Services Research, University Medical Center Hamburg-Eppendorf, Hamburg, Germany; 3 Department of Neurosurgery, Berufsgenossenschaftliche Kliniken Bergmannstrost, Halle (Saale), Germany; 4 Department of Neurosurgery, Klinikum St. Georg gGmbH, Leipzig, Germany; 5 Department of Neurosurgery, University of Leipzig, Leipzig, Germany; 6 Department of Psychiatry and Psychotherapy, University of Leipzig, Leipzig, Germany; 7 Center for Public Mental Health, Gösing am Wagram, Austria; 8 Department of Public Health, University of Cagliary, Cagliary, Italy; McGill University, Canada

## Abstract

**Objective:**

To examine rehabilitation preferences, participation and determinants for the choice of a certain rehabilitation setting (inpatient vs. outpatient) and setting-specific rehabilitation outcomes.

**Methods:**

The longitudinal observational study referred to 534 consecutive disc surgery patients (18–55 years). Face-to-face baseline interviews took place about 3.6 days after disc surgery during acute hospital stay. 486 patients also participated in a follow-up interview via telephone three months later (dropout-rate: 9%). The following instruments were used: depression and anxiety (Hospital Anxiety and Depression Scale), pain intensity (numeric analog scale), health-related quality of life (Short Form 36 Health Survey), subjective prognosis of gainful employment (SPE-scale) as well as questions on rehabilitation attendance, return to work, and amount of sick leave days.

**Results:**

The vast majority of patients undergoing surgery for a herniated disc attended a post-hospital rehabilitation treatment program (93%). Thereby two-thirds of these patients took part in an inpatient rehabilitation program (67.9%). Physical, psychological, vocational and health-related quality of life characteristics differed widely *before* as well as *after* rehabilitation depending on the setting. Inpatient rehabilitees were significantly older, reported more pain, worse physical quality of life, more anxiety and depression and a worse subjective prognosis of gainful employment before rehabilitation. Pre-rehabilitation differences remained significant after rehabilitation. More than half of the outpatient rehabilitees (56%) compared to only one third of the inpatient rehabilitees (33%) returned to work three months after disc surgery (p<.001).

**Conclusion:**

The results suggest a “pre-selection” of patients with better health status in outpatient rehabilitation. Gaining better knowledge about setting-specific selection processes may help optimizing rehabilitation allocation procedures and improve rehabilitation effects such as return to work.

## Introduction

In Germany, medical rehabilitation predominantly takes place in inpatient rehabilitation (IPR) settings. Outpatient rehabilitation (OPR) is offered comparatively less often [1]. Nevertheless, during the last couple of years many efforts have been made to consolidate local OPR according to the principle “OPR has precedence over IPR” [2], [3]. This development results from the attempt to reduce costs within the public health system [3]. Due to flexible time schedule and proximity to home, OPR permits patients to maintain their job activities, facilitates the involvement of relatives and enables the networking with other services of the healthcare and social welfare system [4]. Furthermore, the focal point of OPR is on functional rehabilitation with a strong emphasis on physical and physiotherapeutic treatments rather than on psychosocial, social-medical or secondary preventive measures. On the other hand, IPR settings provide all-day medical surveillance, as well as comprehensive, interdisciplinary rehabilitation treatments including psychosocial support [4].

In Germany, musculoskeletal disorders such as disc related diseases constitute the largest indication group for medical rehabilitation [5]. Every tenth patient in a general medical practice and every second patient in a specialist practice is seeking help due to a disc related disease [6]. Intervertebral disc diseases are most common in middle-aged patients [6], [7] and therefore mostly concern a period of life that is characterized by employment. Although surgical treatment of a herniated disc is a last resort it is necessary for a certain percentage of patients (about 15%) [8]–[10]. After disc operation, a post-hospital curative treatment period varying from four to six weeks is recommended [11]. In the German healthcare system post-operative rehabilitation treatments are held in either inpatient (IPR) or outpatient (OPR) settings and comprise physical convalescence and rest, gradual enhancement of physical activity and accompanying physiotherapy [11].

Yet little is known about the coherence of rehabilitation success and the chosen rehabilitation setting in patients who have undergone herniated disc surgery. Gaining patient-oriented knowledge about setting-specific selection and allocation processes in such an important patient group may have important influence on developing individually tailored setting recommendations and counseling and therefore help improve rehabilitation effects and enable an easier return to working life.

The following questions will be addressed:

1. What are the rehabilitation preferences of herniated disc surgery patients regarding generally attending rehabilitation and the choice for a specific rehabilitation setting (IPR vs. OPR)?
2. How do IPR participants differ from OPR participants at the time of the acute hospital stay before rehabilitation and what are determinants for the choice of a certain rehabilitation setting?
3. Are there differences in rehabilitation outcomes regarding inpatient and outpatient rehabilitation settings?

## Methods

### Study design

**Figure 1:**
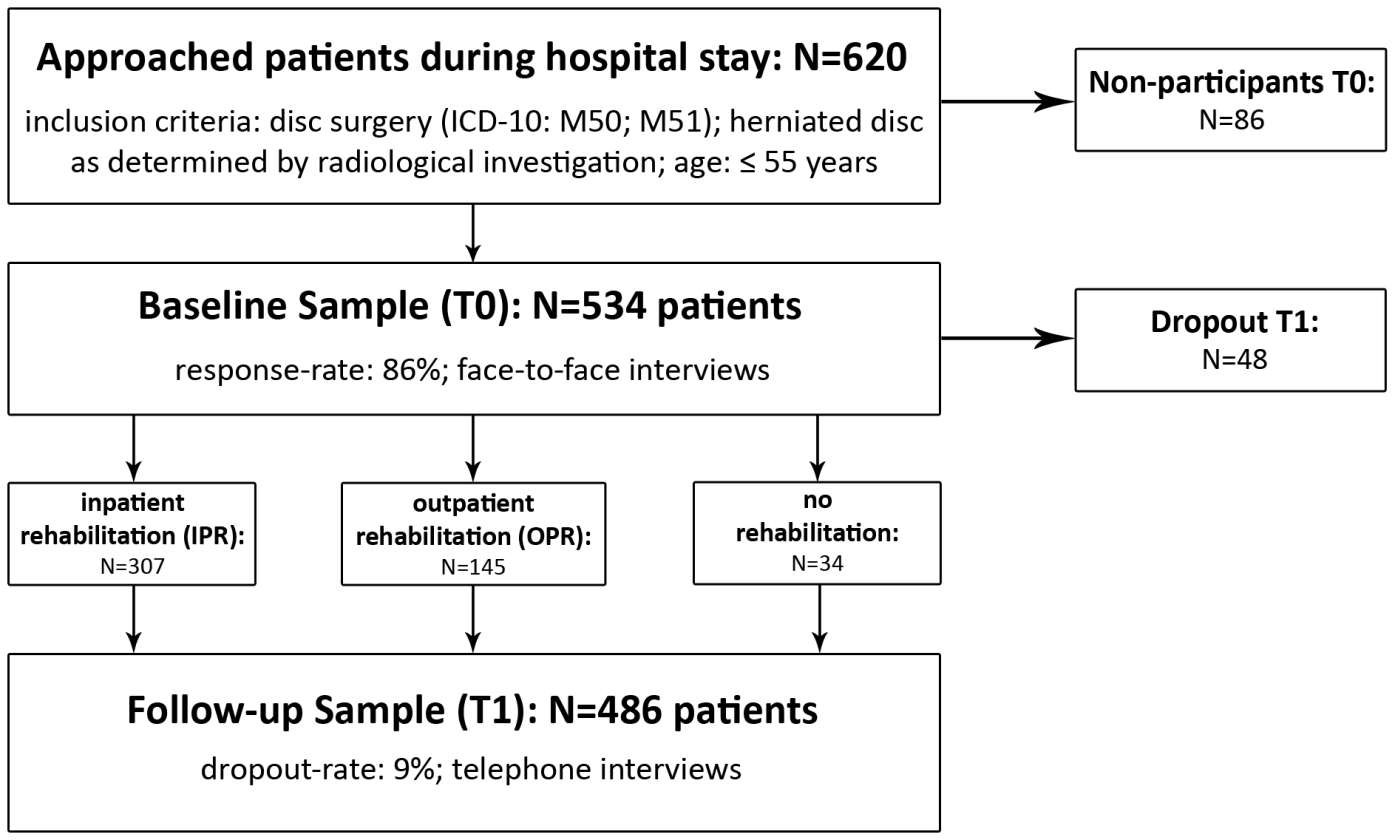
Study design and sample characteristics.

This prospective cohort study refers to two measuring points (figure 1). Baseline interviews (T0) were held face-to face by trained psychologists in acute care hospital about 3.6 days (SD 2.48) after nucleotomy. Follow-up phone interviews (T1) took place three months later.

### Sample

Between April 2007 and October 2008, 620 consecutive nucleotomy patients (figure 1) of three neurosurgery departments in Central Germany were asked to participate in this study. With a response rate of 86%, 534 disc surgery patients finally took part in the baseline interviews (Hospital St. Georg Leipzig (N = 153), University Hospital Leipzig (N = 150), Hospital Bergmannstrost Halle (Saale) (N = 231)). Inclusion criteria in this study were as follows: age between 18 and 55 years, M50 or M51 diagnosis in accordance with International Classification of Diseases (ICD-10) [12], determination of a herniated disc by radiographic examination, condition suitable for rehabilitative care and sufficient knowledge of the German language. A telephone follow-up interview (T1) was held with 486 disc surgery patients (dropout rate 9%).

### Ethics Statement

The study has received ethics committee approval of the University of Leipzig (Ethik-Kommission an der Medizinischen Fakultät der Universität Leipzig). At the initial contact, participants were verbally informed on the purpose of the study (including handing out a study information form) and provided written consent to take part in the study.

### Baseline characteristics (T0) – before rehabilitation

Patients were asked about *socio-demographic variables* such as age, gender, marital status, educational level and whether they had children below the age of 18 years.

*Psychological characteristics* were assessed with the following assessment instruments: Composite International Diagnostic Interview (CIDI-DIA-X, computerized version) [13] and the Hospital Anxiety and Depression Scale – German Version (HADS) [14], [15]. With the fully structured and standardized diagnostic interview CIDI-DIA-X patients' 4-week and 12-months prevalence rates of psychiatric comorbidity were assessed according to the international classification systems of ICD-10 [12] and DSM-IV [16] (selected sections: affective, anxiety and substance related disorders). The HADS is a dimensional measure and self-report rating scale of depression and anxiety consisting of 14 items, each rated on a four-point Likert scale (range 0–3). Higher scores designate more depression and anxiety respectively. Cut-off values to determine anxiety (score of 11 or more) and depression (scores of 9 or more) were chosen in accordance with a representative study of the German adult population [17].

Patients were asked about *illness related variables* such as herniated disc location (lumbar vs. cervical), number of herniated discs and disc surgeries, the existence of other chronic diseases, length of acute hospital stay at the time of disc surgery (medical records) and pain intensity (verbal numeric pain scale (0–100): “If 0 is no pain and 100 is the worst, please give me a number that indicates the amount of pain you are having today.”; higher scores indicate higher pain intensity).

The Short Form 36 Health Survey (SF-36) was used for the assessment of *health-related quality of life* [18] known as a reliable, valid and responsive measurement [19]–[22]. The 36 items of this instrument form eight subscales: physical functioning, role limitations due to physical health problems, bodily pain, general health, vitality, social functioning, role limitations due to emotional problems, and mental health [18]. The eight SF-36-subscales can be aggregated into two distinct summary scores presenting global ratings of mental and physical health (range 0–100, higher scores indicate better quality of life), which is used in the analyses of this study [18].

Study participants were also asked for the following *vocational variables*: employment status, receiving early retirement pension, amount of sick leave days within the last three months, subjective prognosis of gainful employment (SPE-scale: 3 items; score range: 0–3; higher scores represent worse prognosis) [23], [24].

*Rehabilitation related variables* assessed rehabilitation experience in past, whether patients would like to attend rehabilitation after disc surgery and which rehabilitation setting (inpatient vs. outpatient) they would prefer.

### Follow-up characteristics (T1) – after rehabilitation

Patients were asked about their actual *rehabilitation attendance*, and the actual *rehabilitation setting* (inpatient vs. outpatient).

Rehabilitation outcome measurement was related to *psychological well-being* (HADS [14]; [15]), *physical well-being* (disc-related ailments, recurrent disc herniation or disc surgery, pain intensity by using the verbal numeric pain scale (0–100)), *health-related quality of life* (SF-36 [18]) and r*eturn to the work place* (return to work, amount of sick leave days within the last three months, subjective prognosis of gainful employment (SPE-scale [23], [24]).

### Statistical Methods

Statistical analysis were performed using the Statistical Package for the Social Sciences (SPSS) Version 20.0 [25] and STATA Version 12 [26]. Differences before and after rehabilitation regarding the two patient groups IPR/OPR were analysed via chi-square tests and independent t-tests as appropriate. As anxiety, depression, pain intensity, physical and mental quality of life, the subjective prognosis of employment and days of sick leave were measured pre and post rehabilitation repeated measure analyses of variance (ANOVA) were performed to compare the scores between groups (group difference) and within groups (time difference in each group) over time. Independent t-tests were used for post-hoc analyses between groups, Wilcoxon tests for paired samples were used for post-hoc analyses within groups. A two-tailed significance level was set at a = 0.05. Bonferroni corrections were applieded to reduce type I error in interpreting the data. The choice of rehabilitation setting (inpatient vs. outpatient) was analysed using binary logistic regression models. Here, the variables were entered into the model by using two different methods. The enter method allows to include all variables in the logistic regression regardless of a significant effect (full model: FM); in contrast, the forward stepwise method only takes into account variables with a statistically significant contribution (parsimonious model: PM). Besides gender, educational level and disc localization, only variables which differed significantly (after bonferroni corrections) within the Chi-square tests, independent T-tests and repeated measure ANOVA were used as predictors in the regression analyses. Cohen's d, Cramer's V and Partial Eta-squared (η_p_^2^) were used as measures for effect sizes and applied as appropriate for each statistical test. Effect sizes were interpreted as follows: Cohen's d: small (0.2), medium (0.5), large (0.8); Cramer's V: small (0.10), medium (0.30), large (0.50); Partial Eta-squared (η_p_^2^): small (0.01), medium (0.06), large (0.14) [27], [28].

## Results

### Rehabilitation preferences

95.1% of the disc surgery patients preferred to take part in a subsequent rehabilitation at the time of their acute hospital stay. 3.8% of the patients declined participation in rehabilitation. 1.1% of the patients were still uncertain about rehabilitation participation at that time.

61.4% of the patients intending or still uncertain about rehabilitation participation favoured an IPR setting, 36.6% preferred an OPR setting. 2% of the patients did not report a specific preference for a certain rehabilitation setting.

### Actual rehabilitation participation

Within a three months period after the baseline interview, 93.0% of the patients eventually took part in rehabilitation, while 7% did not attend a rehabilitation program at that time. 67.9% of the patients who participated in rehabilitation were in an IPR clinic and 32.1% in an OPR centre. 3.7% of the patients who favoured an IPR program beforehand actually took part in subsequent OPR 1.7% of these patients did not engage in any rehabilitation. 12.9% of the patients who favoured an OPR program beforehand actually took part in subsequent IPR, and 8% of these patients did not engage in any rehabilitation.

### Setting-specific differences before and after rehabilitation

OPR patients more often had children below the age of 18 years, were more often single, had a shorter length of acute hospital stay and had participated less often in rehabilitation in their past at Baseline assessment (table 1). However, according to bonferroni correction (significance level at 0.004) these differences did not remain significant. Applying bonferroni corrections for multiple testings, only younger age was significantly associated with OPR (p<.004), though the effect size (Cohen's d = .343) is rather small. In addition, regression (table 2 and table 3) also revealed younger age at Baseline as a significant determinant for attending an OPR (FM: OR 0.96; p<.05; PM: OR 0.97; p<.05). Also, both regression models (table 2 and table 3) showed a significant association between a better physical health status (SF-36) at Baseline and attending OPR (FM: OR 1.03; p<.05; PM: OR 1.05; p<.01). Regression analysis (table 3) also revealed a lower depression level at Baseline as a significant predictor for attending an OPR (PM: OR 0.93; p<.05).

**Table 1:** Comparison of inpatient rehabilitation participants and outpatient rehabilitation participants ***before*** rehabilitation.

Variables (at T0)		IPR patients (N = 307)			OPR patients (N = 145)					
		mean (SD)	N	%	mean (SD)	N	%	*p*	Cramer's V	Cohen's d
gender	female		139	45.3		54	37.2	.107	.076	
	male		168	54.7		91	62.8			
age		43.4 (7.3)			40.7 (8.4)			**.001**		.343
marital status	single		70	22.8		52	35.9	.007	.149	
	married		191	62.2		69	47.6			
	separated/divorced/widowed		46	15.0		24	16.6			
educational level	till 9th grade		32	10.4		13	9.0	.135	.094	
	10th grade		214	69.7		91	62.8			
	Abitur/Technical college qualification/University qualification		61	19.9		41	28.3			
having children under 18 years	no		198	64.5		74	51.0	.006	.128	
	yes		109	35.5		71	49.0			
psychiatric comorbidity (last 12 months, CIDI)	no		245	80.3		118	81.4	.792	.012	
	yes		60	19.7		27	18.6			
disc location	lumbar		250	81.4		114	78.6	.481	.033	
	cervical		57	18.6		31	21.4			
number of herniated discs	one		181	61.8		93	67.4	.448	.061	
	two		80	27.3		30	21.7			
	three or more		32	10.9		15	10.9			
number of disc surgeries	one		244	81.9		124	88.6	.197	.086	
	two		42	14.1		13	9.3			
	three or more		12	4.0		3	2.1			
other chronic diseases	no		177	57.7		90	62.1	.373	.042	
	yes		130	42.3		55	37.9			
length of acute hospital stay		9.1 (2.6)			8.5 (2.3)			.016		.244
employment status	Fulltime or parttime (min. 15 h/week)		243	79.2		111	76.6	.175	.088	
	Minijob (max.14 h/week) or umemployment		50	16.3		21	14.5			
	Other		14	4.6		13	9.0			
early retirement	no		293	95.4		141	97.2	.361	.043	
	yes		14	4.6		4	2.8			
rehabilitation participation in medical history	no		171	55.7		98	67.6	.016	.113	
	yes		136	44.3		47	32.4			

Calculations via Chi-square-tests and independent T-Tests;

SD: standard deviation; p: p-value;

IPR: inpatient rehabilitation; OPR: outpatient rehabilitation;

CIDI: Composite international diagnostic interview.

Cramer's V and Cohen's d as measures for effect sizes.

Significance level at 0.004 (0.05 divided by 14), bonferroni correction.

**Table 2:** Binary logistic regression model of significant factors associated with the participation in outpatient rehabilitation^a^ (full model).

Baseline (T0) Predictors		B	*OR*	95% CI	p	
**(1) Socio-demographic variables**						
gender	R: female					
	male	.217	1.24	.75–2.06	.399	
age		−.038	.96	.93–1.00	**.029**	*
educational level	R: till 9th grade					
	10th grade	−.182	.83	.29–2.37	.733	
	Abitur/Technical college qualification/University qualification	.111	1.12	.37–3.41	.844	
**(2) psychological variables**						
anxiety (HADS)		−.018	.98	.90–1.07	.674	
depression (HADS)		−.033	.97	.89–1.06	.463	
**(3) illness-related variables**						
disc location	R: lumbar					
	cervical	.535	1.71	.93–3.12	.082	
pain intensity (pain scale)		−.009	.99	.98–1.00	.147	
days of sick leave within the last 3 months		−.002	1.00	.99–1.01	.761	
**(4) health-related quality of life**						
physical health summary scale (SF-36)		.033	1.03	1.00–1.07	**.048**	*
**(5) vocational characteristics**						
subjective prognosis of gainful employment (SPE-scale)		−.169	.85	.62–1.15	.289	

a Reference category is the participation in inpatient rehabilitation.

* p<.05; Nagelkerke's R^2^–0.125.

B: Regression Coefficient B; OR: Odds Ratio; 95% CI: 95% Confidence Interval (OR); *p*: *p*-value; HADS: Hospital anxiety and depression scale; SF-36: Short Form 36; SPE: Subjective prognosis of gainful employment.

**Table 3:** Binary logistic regression model of factors associated with the participation in outpatient rehabilitation^a^ (parsimonious model).

Predictors	Model I				Model II				Model III			
	B	OR	95% CI	*p*	B	OR	95% CI	*p*	B	OR	95% CI	*p*
physical health summary scale (SF-36)	.049	1.05	1.02–1.08	**.001** **	.045	1.05	1.02–1.08	**.003** **	.045	1.05	1.02–1.08	**.003** **
depression (HADS)					−.077	.93	.87–.98	**.010** *	−.069	.93	.88–.99	**.023** *
age									−.036	.97	.93–1.00	**.032** *
**Nagelkerke's R^2^**	.042				.069				.086			

a Reference category is the participation in inpatient rehabilitation.

* p<.05,

** p<.01,

B: Regression Coefficient B; OR: Odds Ratio; 95% CI: 95% Confidence Interval (OR); *p*: *p*-value.

Table 4 shows the results from repeated measure analyses of variance at Baseline (T0) and 3-month follow-up (T1). Accordingly, outcomes such as anxiety, depression, pain intensity, physical health status, subjective prognosis of employment and days of sick leave within the last three months were significantly different (significance level at 0.007, bonferroni correction) between groups (OPR vs. IPR patients). While the effect size for days of sick leave (η_p_^2^ = .095) represents a noticeable medium effect, only small effect sizes for anxiety (η_p_^2^ = .023), depression (η_p_^2^ = .026), pain intensity (η_p_^2^ = .031), physical health status (η_p_^2^ = .043) and subjective prognosis of employment (η_p_^2^ = .037) were found. Post-hoc analyses showed that IPR patients had significantly higher depression and anxiety levels, significantly higher pain intensity, a significantly worse physical health status and a significantly worse subjective prognosis of gainful employment at both assessment points (significance level of 0.05). However, post-hoc analysis revealed that IPR patients only showed significantly more days of sick leave at the 3-month follow-up (T1), not at Baseline (T0). In addition IPR patients were significantly less likely to return to work than OPR patients (table 5, significance level at 0.01, bonferroni correction), even though the effect size is rather small (Cramer's V = .215).

**Table 4:** Repeated measure ANOVA for outcome variables with two assessment points.

	IPR patients	OPR patients						
	Mean (SD)	Mean (SD)	Group Difference		Time Difference		Group × Time Difference	
			p-value	η_p_^2^	p-value	η_p_^2^	p-value	η_p_^2^
Anxiety (HADS)								
Baseline (T0)	7.7 (4.6)	6.6 (4.0)	**.001**	.023	**.000**	.195	.498	.001
3-month follow-up (T1)	5.7 (4.1)	4.3 (3.6)						
Depression								
Baseline (T0)	6.6 (4.5)	5.5 (3.7)	**.001**	.026	**.000**	.202	.605	.001
3-month follow-up (T1)	4.5 (4.0)	3.2 (3.4)						
Pain intensity (pain scale)								
Baseline (T0)	34.2 (24.3)	26.1 (21.5)	**.000**	.031	.842	.000	.989	.000
3-month follow-up (T1)	34.4 (26.3)	26.4 (26.3)						
Physical health summary scale (SF-36)								
Baseline (T0)	26.6 (7.9)	28.6 (7.6)	**.000**	.043	**.000**	.492	.010	.015
3-month follow-up (T1)	36.3 (9.6)	41.1 (11.3)						
Mental health summary scale (SF-36)								
Baseline (T0)	50.7 (12.9)	52.9 (11.1)	.011	.014	**.000**	.033	.371	.002
3-month follow-up (T1)	52.5 (12.5)	55.8 (9.7)						
Subjective prognosis of employment								
Baseline (T0)	1.2 (1.1)	0.9 (1.0)	**.000**	.037	.676	.000	.036	.010
3-month follow-up (T1)	1.3 (1.2)	0.8 (1.0)						
Days of sick leave within the last 3 months								
Baseline (T0)	26.9 (20.4)	22.4 (20.0)	**.000**	.095	**.000**	.426	**.000**	.060
3-month follow-up (T1)	53.9 (16.1)	37.1 (20.5)						

IPR: inpatient rehabilitation; OPR: outpatient rehabilitation.

SD: standard deviation; *p*: p-value; HADS: Hospital anxiety and depression scale; SF-36: Short Form 36; SPE: Subjective prognosis of gainful employment.

Partial Eta-squared (η_p_^2^) as a measure for effect size.

Significance level at 0.007 (0.05 divided by 7), bonferroni correction.

**Table 5:** Comparison of outcome variables with one assessment point (after rehabilitation).

rehabilitation outcome variables (at T1)		IPR patients (N = 307)			OPR patients (N = 145)				
		mean (SD)	N	%	mean (SD)	N	%	*p*	Cramer's V
disc-related ailments	no		141	47.2		72	50.7	.486	.033
	yes		158	52.8		70	49.3		
recurrent disc herniation	no		288	95.0		136	93.8	.581	.026
	yes		15	5.0		9	6.2		
recurrent disc surgery	no		293	95.4		142	97.9	.194	.061
	yes		14	4.6		3	2.1		
return to work (3 months after surgery)	no		205	66.8		64	44.1	**.000**	.215
	yes		102	33.2		81	55.9		

Calculations via Chi-square-tests; SD: standard deviation; *p*: p-value.

IPR: inpatient rehabilitation; OPR: outpatient rehabilitation.

Cramer's V as a measure for effect sizes.

Significance level at 0.01 (0.05 divided by 4), bonferroni correction.

Furthermore the results from repeated measure analyses (table 4) of variance show significant time differences between Baseline (T0) and 3-month follow-up (T1) regarding anxiety, depression, physical health status, mental health status and days of sick leave within the last three months (significance level at 0.007, bonferroni correction). While the effect size for the mental health status was rather small (η_p_^2^ = .033), strong effects were found regarding time differences for anxiety (η_p_^2^ = .195), depression (η_p_^2^ = .202), physical health status (η_p_^2^ = .492) and days of sick leave within the last three months (η_p_^2^ = .426). In the cases of significant time differences, post-hoc analyses were performed. Post-hoc analyses revealed significant improvements (significance level of 0.05) regarding anxiety and depression levels, physical and mental health within both groups (IPR and OPR). In both groups the amount of days of sick leave significantly increased (significance level of 0.05) within post-hoc analyses when comparing the results from Baseline (T0) and 3-month follow-up (T1).

## Discussion

The majority of patients undergoing surgery for a herniated disc attended a post-hospital rehabilitation treatment program. Here two-thirds of these patients took part in an IPR program. Only 7% of the patients did not participate in rehabilitation at all. The presented findings outline that there is almost no difference between the setting-specific rehabilitation preferences during acute hospital stay and the actual rehabilitation participation afterwards. More precisely, most of the patients who wish for IPR at the time of their hospital treatment will in fact attend IPR. The same applies for the preference of an OPR program.

Unlike the results of Woischneck et al. [11], our study sample differed greatly in several socio-demographic, psychological, illness-related and vocational parameters before rehabilitation, depending on the chosen rehabilitation setting. OPR patients were significantly younger, confirming the results of Wolf et al. [29]. In addition, they had more often children below the age of 18 years, even though this result did not remain significant after bonferroni correction of multiple testing. The decision for an IPR setting might be impossible for some patients, as their presence at home may be required for taking care of their young children. Even though the age effect was of small size according to Cohen's conventions [17], [28], regression analysis also revealed younger age as a significant determinant for the choice of an OPR. Younger age might also be associated with the fact of having young children and therefore abet the decision for nearby OPR. Another reason for younger patients to attend OPR might be a better overall physical health status and a lower risk for multimorbidity [30]. This assumption is supported by the finding that younger patients were significantly less likely to have other chronic diseases (Odds Ratio 1.05; p<.001) in our patient sample. Thus, better physical fitness might facilitate dealing with the strain caused by daily driving to OPR. Assumingly, younger patients' expectations and motivations toward rehabilitation might also rather match with the conditions of an OPR. In this context further studies on the impact of rehabilitation expectations and motivations on the choice for a certain rehabilitation setting are strongly recommended.

While Wolf et al. [29] found significantly higher OPR participation rates in women, no gender-specific setting differences were revealed in the presented study. Our study could also not replicate the results of Merkesdal et al. who showed a significantly higher educational level in OPR patients compared to inpatient rehabilitees [31].

Regarding illness-related characteristics such as disc location, the number of herniated discs and surgeries in medical history, no setting-specific differences could be found before rehabilitation. The presence of other chronic diseases was also not associated with the choice for a specific rehabilitation setting, which is contradictory to the results of Wolf et al. [29]. Although the effect was small, inpatient rehabilitees reported a significantly higher pain intensity (p<.01) before rehabilitation than outpatient rehabilitees. Wolf et al. [29] also found lower pain intensity before rehabilitation in back patients participating in OPR compared to IPR patients. These results indicate that disc surgery patients attending IPR experience more physical impairment prior to rehabilitation than OPR patients. The significantly worse physical health-related quality of life in inpatient rehabilitees prior to rehabilitation provides even more evidence for the assumption that inpatient rehabilitees have a worse health status compared to outpatient rehabilitees before rehabilitation, confirming results of Wolf et al. [29]. These results can be confirmed by multivariate analysis, which showed that a better physical health status at Baseline is associated with a subsequent participation in OPR. Complementarily, IPR patients also reported more often rehabilitation experiences in the past, even though this result does not remain significant after bonferroni correction of multiple testing. Nevertheless, this result could be an indicator for a supposably worse physical state of health. Moreover, IPR patients also suffer from more psychological distress prior to rehabilitation, which is indicated by significantly higher anxiety and depression scores compared to outpatient rehabilitees. Even though these effects were rather small, regression analysis could also show that a lower depression level at Baseline is significantly associated with a subsequent participation in OPR.

IPR patients also have a significantly worse prognosis to stay in a job at the time of acute hospital stay than OPR patients. Assuming that IPR patients experience stronger physical and mental impairments before rehabilitation, a worse subjective prognosis of gainful employment might derive from vocational discouragement due to health problems.

Regarding rehabilitation outcome three months after disc surgery, it becomes clear that the pre-rehabilitation differences regarding psychological, illness-related and vocational parameters depending on the chosen rehabilitation setting remain stable in the study sample of herniated disc patients.

Occupational activity is an essential part of our living environment [32]. Regarding vocational rehabilitation, outcome variables in the presented study reveal that OPR patients return to the work place significantly more often than IPR patients. Whereas more than half of the outpatient rehabilitees (56%) return to work three months after disc surgery, it is only one third of the inpatient rehabilitees (33%). This effect is small, but goes well in line with the findings of Woischneck et al. [11], who found significantly higher return to work rates in OPR patients six months after disc surgery. Again, a possible explanation for the extraordinarily lower return to work rates in IPR patients three months after disc surgery can be seen in the worse overall health status, including physical and psychological aspects. Thus, OPR patients report significantly lower pain intensity, better physical quality of life and less anxiety and depression than IPR patients three months after disc surgery. In line with this, a medium-sized effect is reported for the amount of sick leave days within the last three months (time between surgery and follow-up interview), which is significantly higher in IPR patients.

These findings should not be interpreted as quality differences between IPR and OPR treatments. On the contrary, our findings could show that both groups show significant improvements regarding depression, anxiety, physical and mental health status three months after disc surgery. This improvement is underlined by strong effect sizes. Taking into account that setting-specific group differences were already prevalent before rehabilitation treatment, these findings rather suggest a “pre-selection” of herniated disc patients with better health status in OPR and with poorer health status in IPR settings [9]. Accordingly, patients might tend to choose a rehabilitation setting which is assumedly best for their medical condition. Patients with a poor health status might associate IPR with a better benefit for their health based on the assumption of a less stressful and more intense treatment. Patients with a better state of health might rather take social considerations into account by choosing an OPR setting in their home environment and also feel comfortable with the daily drive to their treatment.

### Study Limitations

A limitation of this study is the relatively short period of time when regarding return to work three months after disc surgery [9] as an important indicator for rehabilitation success. An increase of return to work rates in both inpatient and OPR patients must be assumed in a longer follow-up period. Further investigations of the influence of rehabilitation settings on the return to work by using long-term longitudinal study designs are therefore strongly recommended [9]. Another limitation is, that this work does not contain information on preoperative severity of disease such as pain or neurological signs. A greater severity of illness before surgery might also have impacted setting choice and rehabilitation outcome. Complementarily, binary logistic regression analysis only explained 12.5% of the variance (Nagelkerke's R^2^ = 0.125), which implies that there must be other associated factors contributing to the choice for a certain rehabilitation setting. Therefore future studies, that examine the exact allocation procedures for different rehabilitation settings are recommended. Finally, outcome differences might also derive from clinic-specific and not only setting-specific differences, as patients went to different inpatient and outpatient rehabilitation clinics. Event though inpatient and outpatient rehabilitation programs contain the same standard treatments (e.g. physiotherapy, massages, sport therapy, psychological and vocational counselling), there might have been clinic-specific differences. In line with this, motivational differences between individuals might have had an impact on how many treatment offers patients took advantage of. Future studies should take these issues into account.

## Conclusion

The great majority of herniated disc patients participate in rehabilitation after surgery. Our findings indicate a “pre-selection” of disc surgery patients with better health status in OPR and with poorer health status in IPR settings [9]. As a result, the worse rehabilitation outcome of IPR patients must be attributed to their worse pre-rehabilitation health status. More precisely, more impaired and distressed patients before rehabilitation might not benefit as much from their rehabilitation treatment than less impaired and distressed patients.

During the last few years patient orientation and patient participation have gained substantial importance for research and health services [33]. In this context it seems even more important to investigate setting-specific selection processes in disc surgery patients that might be linked to patient's expectations and motivations toward rehabilitation. Gaining better knowledge about these setting-specific selection processes may have major implications for rehabilitation allocation procedures. Optimizing these allocation procedures may be of utmost importance for providing rehabilitation treatments perfectly tailored to the individual's needs.

Additionally, studies on setting-specific differences regarding the long-term rehabilitation outcome are lacking and therefore strongly recommended. Regarding the long-term perspective of rehabilitation outcome by taking setting specific differences into account may help to improve rehabilitation effects.
